# Amygdala subregion MRI alterations in Parkinson’s disease correlate with olfactory function

**DOI:** 10.3389/fnagi.2026.1879832

**Published:** 2026-07-20

**Authors:** Huandi Lv, Xingyu Wang, Ziqi Wang, Lin Liu, Qianhang Yang, Shenao Li, Xuefang Han, Duo Gao, Jiaxin Wang, Yankai Wu, Caixia Cui, Jingjing Cui, Liuyi An, Zuojun Geng

**Affiliations:** 1 Department of Medical Imaging, The Second Hospital of Hebei Medical University, Shijiazhuang, Hebei, China; 2 Department of Radiology, The Affiliated Hospital, Southwest Medical University, Luzhou, Sichuan, China; 3 Precision Imaging and Intelligent Analysis Key Laboratory of Luzhou, Southwest Medical University, Luzhou, Sichuan, China; 4 Department of Research and Development, Shanghai United Imaging Intelligence Co, Ltd., Shanghai, China; 5 Hebei Key Laboratory of Medical Imaging, Shijiazhuang, China

**Keywords:** amygdala subregions, automated segmentation, olfactory dysfunction, Parkinson’s disease, structural MRI

## Abstract

**Objective:**

Olfactory dysfunction affects 80–90% of Parkinson’s disease (PD) patients. The amygdala, a key olfactory node, comprises functionally distinct subregions. We investigated volumetric changes in nine amygdala subregions across PD stages and their relationship with olfactory function.

**Methods:**

In this cross-sectional study, 65 PD patients (40 early stage, Hoehn-Yahr 1.0–2.5; 25 late- stage, 3.0–5.0) and 45 healthy controls underwent high-resolution 3D-T1WI MRI. Automated segmentation quantified bilateral volumes of nine amygdala subregions. Olfactory function was assessed using Sniffin’ Sticks (threshold, discrimination, identification, TDI score). Cognitive function was evaluated by MMSE. Statistical analyses included ANCOVA/GLM with Bonferroni correction and Spearman partial correlation.

**Results:**

PD patients exhibited significant volume reductions in bilateral cortical nucleus (Co), accessory basal nucleus (AB), cortico-amygdaloid transition area (CAT), and right anterior amygdaloid area (AAA) versus controls (*P* < 0.05). Bilateral Co and right AAA were already reduced in early stage PD. Controlling for age, sex, education, and intracranial volume, amygdala subregion volumes showed no statistically significant correlations with olfactory scores, though right AAA volume weakly correlated with threshold (*R* = 0.27) and TDI total score (*R* = 0.24) (*P* > 0.05). Additionally, the volume of the left medial subdivision (Me) of the amygdala showed a significant positive correlation with MMSE scores (*R* = 0.29, *P* = 0.026).

**Conclusion:**

PD involves subregion-selective amygdala atrophy, with Co and AAA affected early, followed by AB and CAT, exhibiting a right-lateralized pattern. The weak correlation between right AAA volume and olfactory function suggests a specific role in PD-related olfactory dysfunction, providing novel insights into its structural basis. Additionally, left Me volume correlated with MMSE independent of stage, implying a structural biomarker for cognition.

## 1 Introduction

Parkinson’s disease (PD) is the second most common neurodegenerative disorder (GBD 2016 Parkinson’s Disease Collaborators, 2018; Su et al., 2025), characterized pathologically by progressive loss of dopaminergic neurons in the substantia nigra pars compacta and abnormal aggregation of α-synuclein (α-syn) forming Lewy bodies (Morris et al., 2024). By the time patients present with classic motor symptoms, approximately 70% of nigral dopaminergic neurons have already degenerated or undergone apoptosis (Braak and Del Tredici, 2008). The prodromal phase is dominated by non-motor symptoms, among which olfactory dysfunction occurs in 80–90% of patients (Doty, 2012) and has been recognized by the International Parkinson and Movement Disorder Society (MDS) as an important prodromal biomarker (Berg et al., 2015; Heinzel et al., 2019). Olfactory dysfunction can persist throughout the course of PD, and most patients are unaware of their impairment (Doty, 2017; Kondo et al., 2020), highlighting the value of olfactory testing for early identification.

In the olfactory pathway, the olfactory bulb receives signals and projects to the primary olfactory cortex (amygdala, entorhinal cortex, piriform cortex, etc.), which then transmits signals to secondary olfactory cortices (hippocampus, orbitofrontal cortex, etc.) (Mori and Sakano, 2021). Regarding the distribution of Lewy pathology (LP) in PD, the Braak hypothesis proposes that LP spreads progressively from the peripheral olfactory nerve to central olfactory cortices (Braak and Del Tredici, 2008; Beach et al., 2009). However, a systematic review by Surmeier indicates that LP is not diffusely distributed but rather selectively appears in specific brain regions, such as the medulla, pons and midbrain, the amygdala (especially its olfactory and basolateral subregions), the prefrontal cortex, and the hippocampal CA2 area. Moreover, only about 50% of PD patients fully conform to the Braak staging (Surmeier et al., 2017). Although the two theories differ in their interpretation of the spreading pattern of LP, both identify the amygdala as one of the commonly affected regions. The amygdala, located deep within the temporal lobe and composed of multiple functionally specialized subregions (Fox and Shackman, 2024; Kim et al., 2024), serves as a key hub connecting the olfactory pathway with emotion, memory, and executive function networks (Li and Wilson, 2024; Liu et al., 2024). Therefore, the amygdala represents an ideal target for investigating PD-related olfactory dysfunction and its associated neural mechanisms.

Previous neuroimaging studies of the amygdala in PD have largely treated it as a single entity or roughly divided it into 2–5 subregions (Damme et al., 2020; Ay et al., 2023). With the development of MRI and automated segmentation techniques, *in vivo* analysis of fine amygdala subregions has become feasible (Liu et al., 2020; Qu et al., 2024).

This study hypothesized that volume changes in specific amygdala subregions in PD patients are specifically associated with olfactory dysfunction and cognitive decline. To test this hypothesis, we conducted a cross-sectional study of 110 subjects aged 43–80 years, using automated segmentation to divide the amygdala into nine fine subregions, analyzing volume changes across different disease stages, and further exploring the relationship between subregional volumes and olfactory and cognitive function.

## 2 Materials and methods

### 2.1 Participants

Sixty-five patients with primary Parkinson’s disease attending the Department of Neurology, Second Hospital of Hebei Medical University from March 2024 to March 2026 were enrolled, including 40 early stage PD (H-Y 1.0–2.5) and 25 late-stage PD (H-Y 3.0–5.0). The Hoehn and Yahr (H-Y) stage grades Parkinson’s disease severity on a scale of 1 (mild) to 5 (severe). Forty-five healthy controls (HC) were recruited during the same period. All subjects provided informed consent, and the study was approved by the hospital ethics committee (2024-R210).

Inclusion criteria, PD group: Diagnosed with primary Parkinson’s disease based on the 2016 Chinese diagnostic criteria for Parkinson’s disease (Parkinson’s Disease and Movement Disorders Group et al., 2016); All patients underwent a 12-h drug washout (OFF state); Right-handed; Complete MRI data with good quality; Able to complete MRI, olfactory testing, and MMSE (Mini-Mental State Examination); No sinusitis, nasal masses, or other conditions affecting olfaction; No history of severe head trauma, surgery, or other organic brain lesions; No other neurological or psychiatric disorders. HC group: Right-handed; No history of systemic diseases; No nasal diseases affecting olfaction; No history of severe head trauma, surgery, or other organic brain lesions; No neurological or psychiatric disorders.

Exclusion criteria: Inability to complete MRI (metal implants, claustrophobia, etc.); Severe image artifacts; Cerebral white matter Fazekas ≥ grade II; Extremely low MMSE scores; Alcohol or drug dependence.

### 2.2 Cognitive assessment

MMSE assessment was performed by a neurologist.

### 2.3 Olfactory testing

Sniffin’ Sticks olfactory test was used, comprising threshold test (TT, 0–16 points), discrimination test (DT, 0–16 points), and identification test (IT, 0–16 points). TDI is the sum of the three scores. Classification: TDI > 30 = normal olfaction, 16–30 = hyposmia, < 16 = anosmia.

### 2.4 MRI acquisition and parameters

MRI was performed using a GE Signa Architect 3.0T MRI scanner with a 48-channel phased-array head coil. Subjects were placed in the supine position with head first, standard head positioning, using the anterior-posterior commissure line as the scanning baseline, scanning from the foramen magnum to the vertex. First, MAGiC sequence scanning was performed to exclude intracranial organic diseases, followed by a 3D-T1WI fast spin echo sequence.

MAGiC sequence (TR = 4,150 ms, TE = 18.6 ms, slice thickness = 4 mm, slice gap = 1 mm, FOV = 240 × 240 mm, matrix = 256 × 256, 30 slices, scan time = 5 min 16 s).

3D-T1WI sequence (TR = 6.2 ms, TE = 2.4 ms, slice thickness = 1 mm, FOV = 256 × 256 mm, matrix = 256 × 256, 160 slices, FA = 8°, scan time = 5 min 41 s).

The United Imaging brain volume measurement software^1^ was used to analyze 3D-T1WI data and automatically extract volumes. This method is based on the FreeSurfer software and employs 3D deep learning segmentation model (Wu et al., 2023). Supplementary material, including the data availability statement and details of integrated open-source library toolkits in the analytical platform, are available online at: https://www.frontiersin.org/articles/10.3389/fradi.2023.1153784/full#supplementary-material. The processing steps included: (1) bias field correction and adaptive normalization of image grayscale; (2) skull stripping; (3) tissue segmentation of white matter, gray matter, and cerebrospinal fluid (CSF); (4) whole-brain bilateral segmentation and subregional segmentation. Volumes of the left and right amygdala and their nine subregions (Lateral nucleus, La, Medial nucleus, Me, Basal nucleus, Ba, Accessory basal nucleus, AB, Central nucleus, Ce, Anterior amygdaloid area, AAA, Cortical nucleus, Co, Cortico-amygdaloid transition area, CAT, Paralaminar nucleus, PL) were automatically calculated.

### 2.5 Statistical analysis

SPSS 27.0 and R Studio were used for statistical analysis, with *P* < 0.05 considered statistically significant.

Sex was analyzed using chi-square test. For continuous variables, normality was tested using Shapiro-Wilk test; normally distributed data were expressed as mean ± SD and analyzed using one-way ANOVA; non-normally distributed data were expressed as median ± interquartile range and analyzed using non-parametric tests. For olfactory scores (TT, DT, IT, TDI), if normality and homogeneity of variance assumptions were met, ANCOVA with age, sex, and education as covariates was performed, followed by Bonferroni correction. Otherwise, generalized linear models (linear, with robust estimation) were used, followed by sequential Bonferroni correction.

Amygdala and Subregion Volumes, With age, sex, education, and total intracranial volume (ICV) as covariates, ANCOVA or generalized linear models were used as above, with the same *post-hoc* correction methods. Specifically, although sex distribution did not differ significantly among the three groups, sex was included as a standard covariate in all volumetric analyses because it is a well-established biological factor influencing amygdala and subregion volumes (Ruigrok et al., 2014; Gui et al., 2025; Overholtzer et al., 2025). Covariate adjustment does not require a strict proportional relationship between regional volume and ICV, is more robust to data deviations, and preserves the original volumetric scale for comparability (O’Brien et al., 2011; Voevodskaya et al., 2014); it is therefore the recommended approach for subcortical structures such as the amygdala (Barnes et al., 2010).

Spearman partial rank correlation analysis, with sex, age, years of education, and total brain volume as control variables, was performed to evaluate the correlations between amygdala and its subregion volumes and TT, DT, IT, and TDI scores, as well as MMSE scores, in patients with Parkinson’s disease.

Spearman partial rank correlation is a non-parametric partial correlation method used to assess the rank-based (monotonic) association between two variables while controlling for one or more covariates. This method does not require assumptions about the distribution of the original variables and is also applicable to categorical variables such as sex, making it suitable for our analysis.

All statistical analyses and visualizations were performed using R Studio (R version 4.4.2). Partial rank correlation analyses were conducted using the pcor.test() function from the ppcor package (version 1.1) with the method parameter set to “spearman.” Heatmaps and scatter plots were generated using the ggplot2 package within the tidyverse framework (version 2.0.0), and further refined and annotated using the ggpubr package (version 0.6.2). For residual scatter plots, marginal density plots were added using the ggMarginal() function from the ggExtra package (version 0.10.1) to display the distribution characteristics of variables along the marginal dimensions.

## 3 Results

### 3.1 General information

Table 1 displays the study’s Demographic, clinical, and baseline neuroimaging characteristics of the three groups. A total of 110 subjects were included: 40 early-stage PD (H-Y 1.0–2.5), 25 late-stage PD (H-Y 3.0–5.0), and 45 healthy controls (HC). No significant difference in sex distribution was found among the three groups (*P* > 0.05). Significant differences were observed in age, education years, and H-Y stage (*P* < 0.05).

**FIGURE 1:**
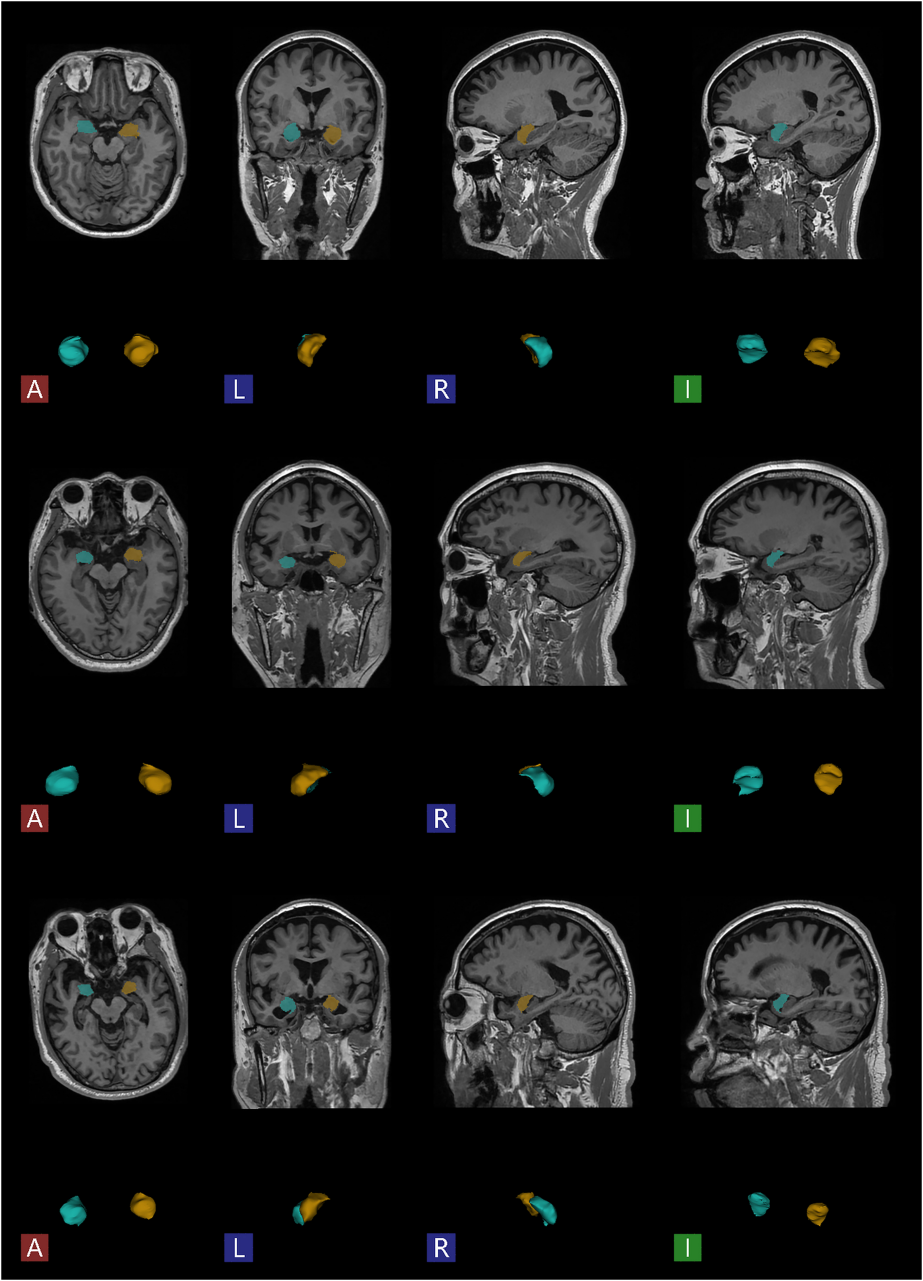
Location and volume of the amygdala. The amygdala (highlighted) is shown in a representative healthy control subject, with its location indicated on the images. This image represents one individual subject, not averaged data.

**FIGURE 2:**
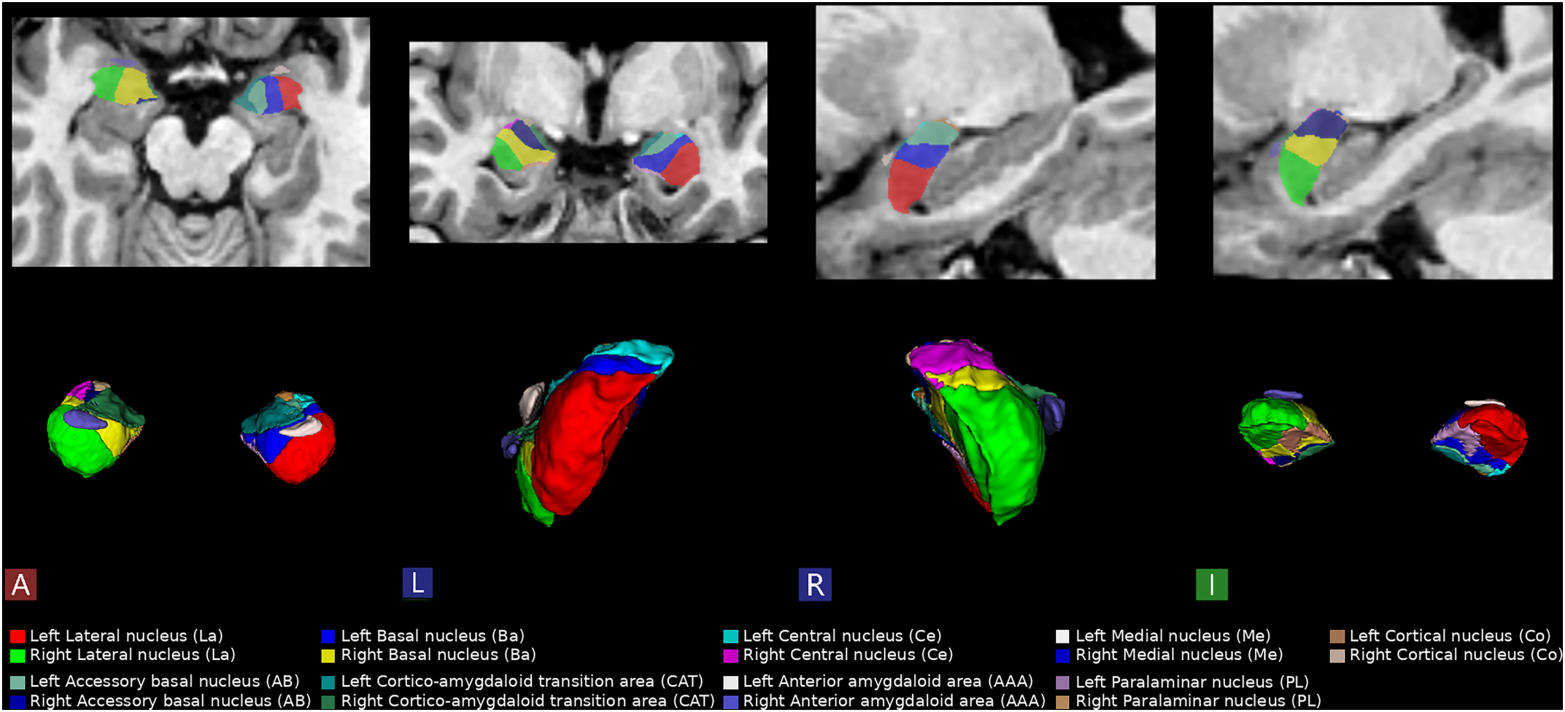
Volume and distribution map of subregions of the amygdala in a representative healthy control subject. The subregions are color-coded for identification. The image is from a single individual subject and does not represent averaged data.

**TABLE 1:** Demographic, clinical, and baseline neuroimaging characteristics of the three groups.

Characteristic	HC (*n* = 45)	ES(*n* = 40)	LS(*n* = 25)	*P*	*P1*	*P2*	*P3*	χ^2^*/F/H*
Age	63.22 ± 6.21	62.3 ± 9.39	67.96 ± 7.68	0.018	0.936	0.034	0.031	4.280
Sex	18/27	20/20	12/13	0.625				0.939
Education				<0.001	0.135	<0.001	0.005	17.503
Primary school or below	4	12	12					
Junior high school	12	13	7					
Senior high school	16	10	5					
College or above	13	5	1					
MMSE	29 (28, 29)	26 (25, 27)	21 (19.5, 24)	<0.001	<0.001	<0.001	0.003	50.741
H-Y stage	—	2 (2, 2.5)	3 (3, 3)	<0.001				50.347
Olfactory								
*T*-test	8.23 ± 0.55	6.86 ± 0.61	5.59 ± 0.60	0.011	0.170	0.010	0.170	8.948
D test	10.08 ± 0.37	8.55 ± 0.35	8.39 ± 0.40	0.005	0.011	0.011	0.750	10.595
I test	11.02 ± 0.41	8.71 ± 0.39	8.09 ± 0.44	< 0.001	<0.001	< 0.001	0.282	24.043
TDI total	29.33 ± 1.08	24.13 ± 1.08	22.07 ± 1.01	<0.001	0.001	<0.001	0.164	19.560

Data for T, D, I, and TDI scores are presented as means ± standard error (SE). Age, analyzed using one-way ANOVA with Welch’s correction, is expressed as mean ± standard deviation (SD). Educational level, H-Y stage, and MMSE score were compared using the Kruskal-Wallis H test and are reported as median (interquartile range, IQR). *P*, values indicate overall differences among the three groups; *P1*, HC-ES Group comparisons; *P2*, HC-LS Group comparisons; *P3*, ES-LS Group comparisons.

MMSE scores: HC > early PD > late PD, with significant differences between groups (*P* < 0.05).

Olfactory function (TT, DT, IT, TDI): HC > early PD > late PD, all differences were statistically significant. Pairwise comparisons showed that except for the TDI score between early and late PD groups (*P* > 0.05), all other intergroup differences were significant (*P* < 0.05).

### 3.2 Amygdala and subregion volume analysis

Figure 1 displays the location and volume of the amygdala, Figure 2 displays the volume and distribution map of subregions of the amygdala. Table 2 displays the findings of the measurement and statistical analysis of the volumes of Amygdala and Subregion. No significant difference in total intracranial volume (ICV) was found among the three groups (*P* = 0.397). Significant overall intergroup differences were observed in AMG (R), Ba (L), Co (L), Co (R), AB (L), AB (R), CAT (L), CAT (R), AAA (L), and AAA (R) (*P* < 0.05).

**TABLE 2:** Amygdala and subregion volume analysis among the three groups.

Index	HC (*n* = 45)	ES (*n* = 40)	LS (*n* = 25)	*P*	*P*1	*P*2	*P*3	*F/Wald χ^2^*
ICV (cm^3^)	1406.96 ± 125.86	1425.63 ± 122.11	1381.66 ± 134.60	0.397				0.931
MG (L)	1.943 ± 0.031	1.893 ± 0.040	1.804 ± 0.054	0.081	0.924	0.083	0.564	2.579
AMG (R)	2.034 ± 0.031	2.018 ± 0.040	1.869 ± 0.055	0.031*	1.000	0.013*	0.088	3.588
La (L)	0.620 ± 0.010	0.601 ± 0.012	0.600 ± 0.017	0.382	0.693	0.937	1.000	0.970
La (R)	0.641 ± 0.009	0.610 ± 0.017	0.604 ± 0.016	0.099	0.240	0.164	0.773	4.626
Ba (L)	0.438 ± 0.006	0.416 ± 0.008	0.418 ± 0.009	0.037*	0.074	0.134	0.835	6.605
Ba (R)	0.462 ± 0.006	0.443 ± 0.007	0.440 ± 0.011	0.089	0.147	0.207	0.841	4.829
Ce (L)	0.034 ± 0.001	0.032 ± 0.001	0.031 ± 0.001	0.140	0.265	0.265	0.824	3.936
Ce (R)	0.035 ± 0.001	0.032 ± 0.001	0.330 ± 0.001	0.108	0.131	0.368	0.669	4.456
Me (L)	0.002 ± 0.000	0.002 ± 0.000	0.002 ± 0.000	0.137	0.637	0.195	1.000	2.024
Me (R)	0.002 ± 0.000	0.002 ± 0.000	0.002 ± 0.000	0.067	0.087	0.493	1.000	2.768
Co (L)	0.020 ± 0.001	0.018 ± 0.001	0.016 ± 0.001	<0.001*	0.018*	<0.001*	0.375	9.076
Co (R)	0.021 ± 0.001	0.019 ± 0.001	0.018 ± 0.001	0.003*	0.024*	0.006*	0.626	11.444
AB (L)	0.252 ± 0.004	0.237 ± 0.005	0.237 ± 0.006	0.043*	0.106	0.106	0.950	6.278
AB (R)	0.262 ± 0.005	0.249 ± 0.006	0.231 ± 0.008	0.005*	0.297	0.004*	0.208	5.687
CAT (L)	0.145 ± 0.003	0.135 ± 0.003	0.137 ± 0.003	0.027*	0.050	0.093	0.702	7.260
CAT (R)	0.151 ± 0.003	0.144 ± 0.004	0.134 ± 0.005	0.008*	0.274	0.008*	0.334	5.089
AAA (L)	0.025 ± 0.001	0.023 ± 0.001	0.021 ± 0.001	0.012*	0.274	0.013*	0.443	4.657
AAA (R)	0.028 ± 0.001	0.026 ± 0.001	0.024 ± 0.001	0.002*	0.014*	0.003*	0.186	12.139
PL (L)	0.031 ± 0.001	0.030 ± 0.001	0.031 ± 0.001	0.692	1.000	1.000	1.000	0.735
PL (R)	0.035 ± 0.001	0.034 ± 0.001	0.034 ± 0.001	0.583	1.000	1.000	1.000	0.543

Total brain volume is presented as mean ± standard deviation (SD); amygdala subregion volumes are presented as estimated marginal means ± standard error (SE); *P*, values indicate overall differences among the three groups; *P*1, HC-ES Group comparisons; *P*2, HC-LS Group comparisons; *P*3, ES-LS Group comparisons.

*P < 0.05, significant between-group volume differences.

Pairwise comparisons revealed: (1) Bilateral Co and right AAA volumes were significantly larger in HC than in both early and late PD groups (Bonferroni correction, *P* < 0.05), with no significant difference between early and late PD groups (*P* > 0.05); (2) Volumes of AMG (R), AB (R), CAT (R), and AAA (L) were significantly larger in HC than in late PD group (Bonferroni or sequential Bonferroni correction, *P* < 0.05), but no significant differences were found between HC and early PD or between early and late PD groups (*P* > 0.05); (3) No significant differences were found between early and late PD groups for any subregion with overall intergroup differences (*P* > 0.05).

### 3.3 Correlation analysis

**FIGURE 3:**
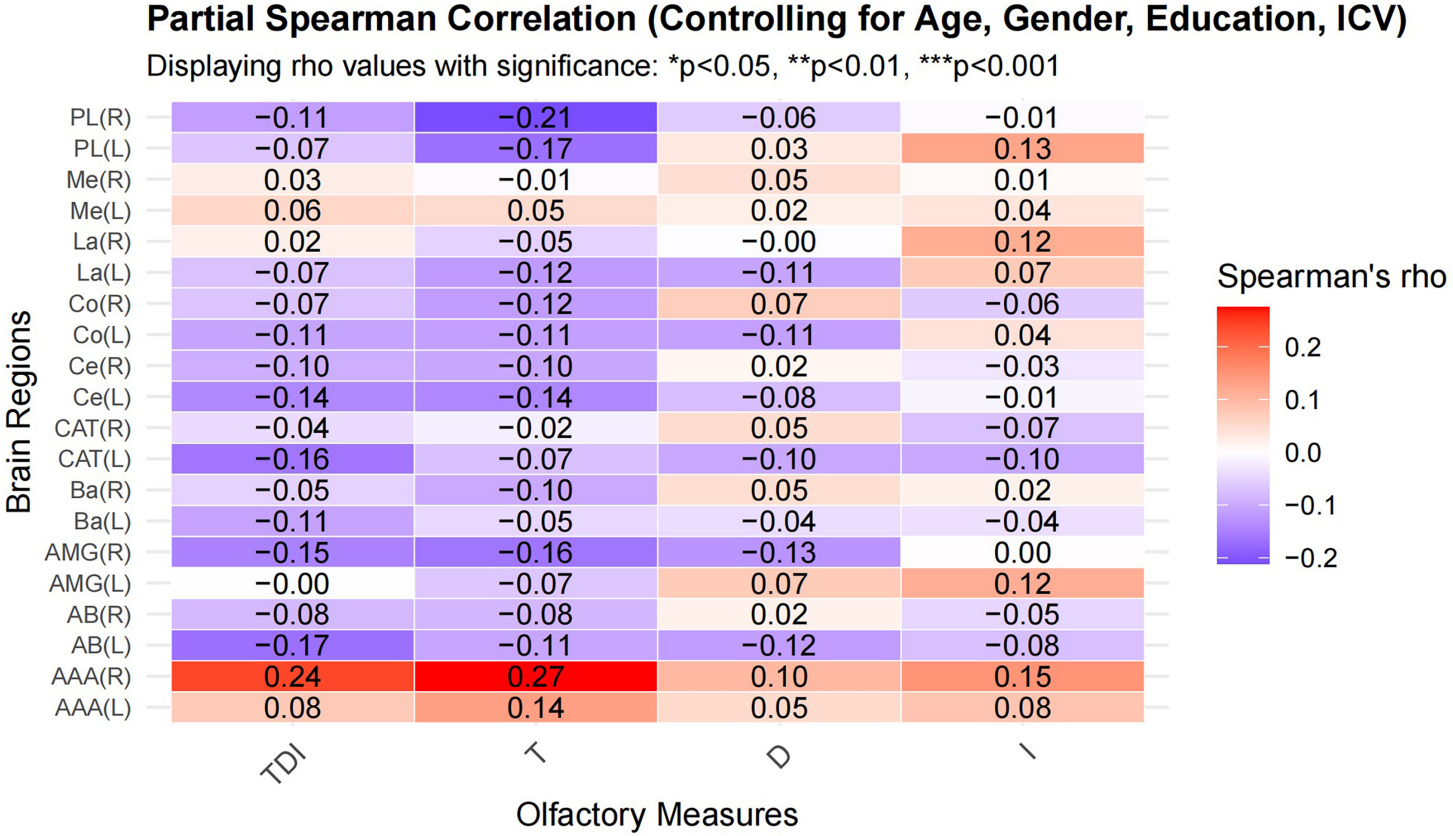
Correlation analysis of amygdala and subregion volumes with olfactory test scores in patients with PD. *P < 0.05, **P < 0.01, ***P < 0.001.

Figure 3 displays the correlation analysis between olfactory function and Amygdala subregion volumes. After controlling for age, sex, education, and total intracranial volume, partial rank correlation analysis showed that right AAA volume in PD patients was weakly correlated with TT (*R* = 0.27) and TDI total score (*R* = 0.24), but the correlations were not statistically significant (*P* > 0.05). The remaining subregions showed very weak or no significant correlations with olfactory test scores.

**FIGURE 4:**
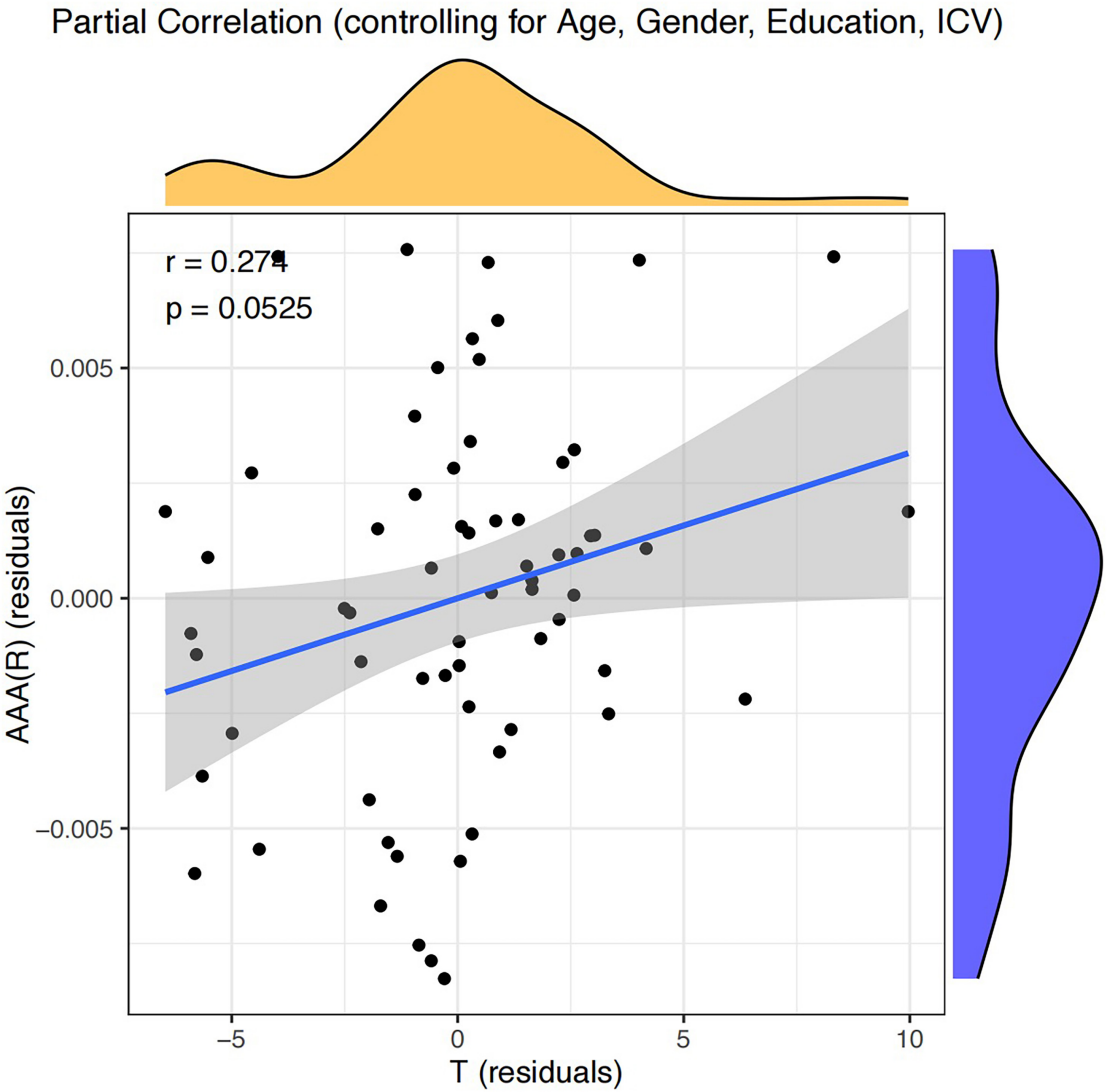
Correlation between AAA-R (right amygdala subregion) and T (Threshold test).

**FIGURE 5:**
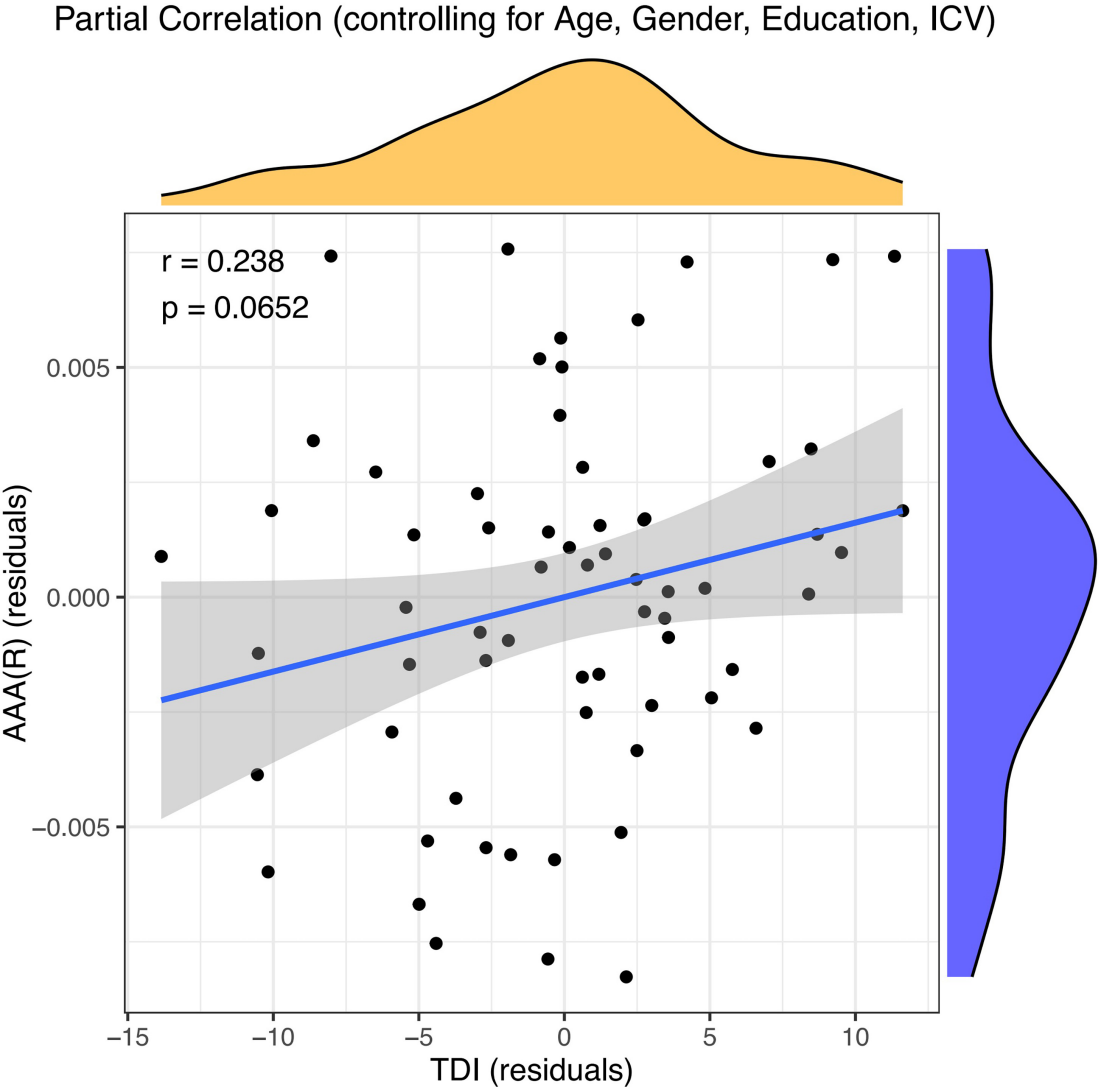
Correlation between AAA-R (right amygdala subregion) and TDI (Threshold, Discrimination, and Identification scores).

Figures 4, 5 display scatter plots illustrating the correlations of right AAA volume with TT (*R* = 0.27, *P* = 0.053) and with TDI (*R* = 0.24, *P* = 0.065) total score, respectively, individual data points are shown, with the linear regression line and the corresponding correlation coefficient and *p*-value indicated.

**FIGURE 6:**
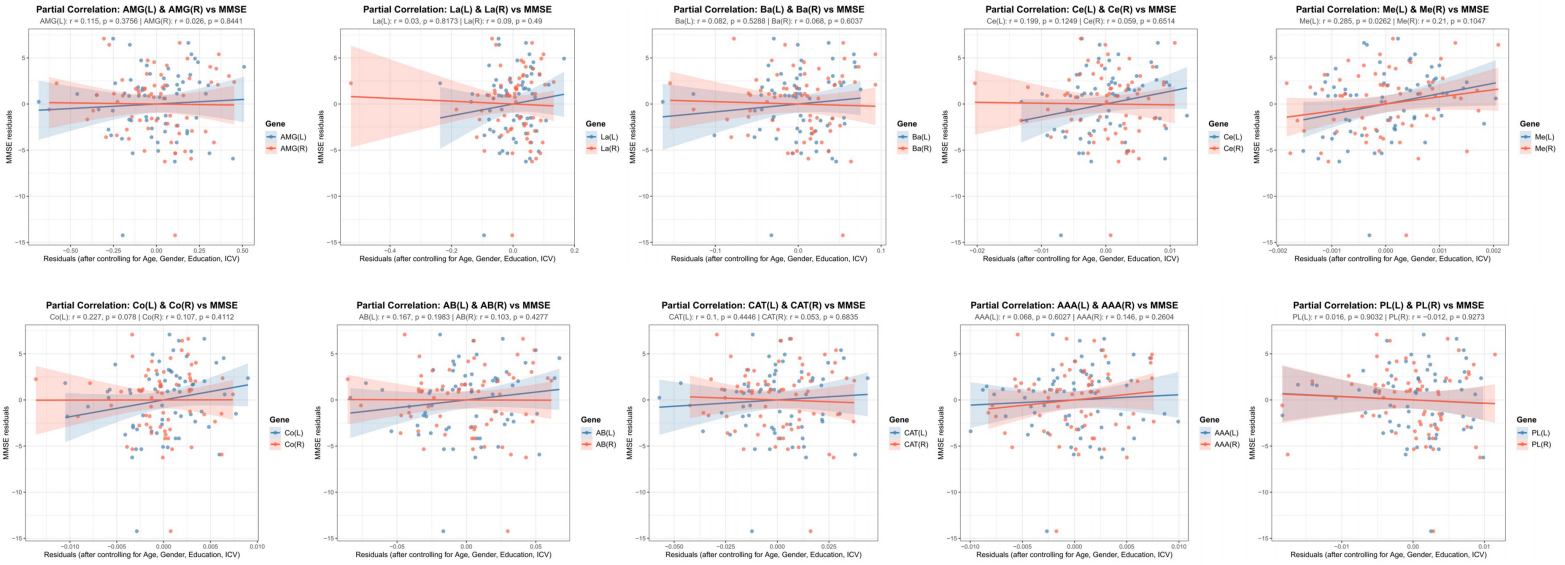
Correlation Analysis of Amygdala and Subregion Volumes with MMSE in Patients with PD.

Figure 6 shows a statistically significant correlation between the volume of the medial subdivision (Me) of the left amygdala and MMSE scores in PD patients (*R* = 0.29, *P* = 0.026).

## 4 Discussion

This study used 3D-T1WI MRI combined with automated segmentation to perform fine-grained volumetric analysis of nine amygdala subregions in PD patients at different disease stages and healthy controls, and explored their relationship with olfactory function. The main findings are discussed below.

MMSE scores showed a decreasing trend: HC > early PD > late PD, suggesting progressive cognitive decline with disease progression, consistent with previous studies (Sivaranjini and Sujatha, 2021). Olfactory function (TT, DT, IT, and TDI total score) decreased sequentially across the three groups, confirming that olfactory dysfunction is one of the most common non-motor symptoms in PD, with severity associated with disease progression (Jeong et al., 2026). This aligns with Braak’s pathological staging theory, in which α-synuclein pathology first affects the olfactory bulb and olfactory pathway, then gradually spreads to the midbrain and cortex (Braak and Del Tredici, 2008). Notably, no statistically significant difference in TDI score was found between early and late PD groups, suggesting that olfactory function is already significantly impaired in the early stage of the disease, followed by a relative plateau phase.

In this study, we performed a detailed volumetric analysis of nine amygdala subregions specifically in relation to olfactory dysfunction in PD, which revealed a subregion-selective pattern of structural atrophy. The results showed significant volume reductions in bilateral Co, AB, CAT, AAA, and Ba (L), as well as the right amygdala overall, in PD patients. Notably, bilateral Co and right AAA were already reduced in early stage PD, while reductions in right amygdala overall, right AB, right CAT, and left AAA reached statistical significance only in late-stage PD. This finding reveals the selective vulnerability and different temporal sequences of involvement of amygdala subregions in the course of PD.

The cortical nucleus (Co) and cortico-amygdaloid transition area (CAT) belong to the superficial nuclear group of the amygdala and have extensive fiber connections with the olfactory cortex and medial temporal lobe structures. The AAA is anatomically an extension of the basolateral amygdala complex, and both receive direct projections from the olfactory bulb (Echevarria-Cooper et al., 2022). According to Braak’s pathological staging theory, these subregions are located relatively upstream in the hierarchical spread of α-synuclein pathology. The finding that bilateral Co and right AAA were already significantly reduced in early stage PD suggests that these subregions are highly sensitive to PD-related pathological changes, consistent with the pathological staging foundation. Ay et al. (2023) also found that bilateral CAT volumes were significantly reduced in PD patients with cognitive impairment, suggesting that CAT is not only a key node in the olfactory pathway but also closely related to cognitive function. In this study, right CAT volume was significantly reduced only in the late stage PD group, which may be related to the more pronounced cognitive decline in late stage patients, and CAT structural changes may reflect disease progression toward the stage of cognitive impairment.

The accessory basal nucleus (AB) and basolateral nucleus (Ba) belong to the basolateral amygdala complex, receiving highly processed information from the secondary olfactory cortex (piriform cortex, orbitofrontal cortex) and participating in emotional valence encoding of olfactory information and emotional-autonomic function integration (Wang et al., 2023). Unlike the superficial nuclei (Co, CAT), which receive direct monosynaptic projections from the olfactory bulb, AB and Ba are located relatively downstream in the hierarchical spread of α-syn pathology according to Braak’s theory, and therefore structural changes appear later. In this study, volume reductions in AB and Ba occurred predominantly in the late-stage PD, mutually corroborating the notion that structural changes in these subregions may be markers of disease progression.

Lateralization phenomenon: Right AAA and right amygdala overall showed significant changes in early or late stages, while left AAA reached significance only in the late- stage group, and right CAT volume reduction also emerged earlier than left. This right lateralization may be related to hemispheric specialization of the olfactory pathway. Studies have shown that human olfactory processing has a right hemisphere advantage, particularly in olfactory emotional processing (Spinella, 2002). This lateralization may render right amygdala subregions more sensitive to olfactory-related pathological changes.

Notably, no significant differences in amygdala subregion volumes were found between early and late PD groups in this study, suggesting that amygdala structural changes may reach a certain degree early in the disease, with relatively slow subsequent progression. However, this finding may also reflect the high heterogeneity of PD in terms of progression patterns and subtypes. As one of the early brain regions affected by α-synuclein pathology, the amygdala may show different degrees and trajectories of involvement depending on the subtype, rather than progressing linearly with clinical stage.

The “brain-first/body-first” hypothesis proposed by Borghammer (2021, 2023) offers a new perspective on PD heterogeneity. According to this hypothesis, PD is divided into two subtypes: in the brain-first subtype, initial pathology begins in the central nervous system, often in the olfactory bulb or amygdala, and then spreads ipsilaterally; in the body-first subtype, initial pathology begins in the enteric nervous system and ascends to the central nervous system via the vagus nerve.

This framework helps explain why amygdala changes do not necessarily progress uniformly with clinical stage. On one hand, the amygdala is one of the initial sites in the brain-first subtype; thus, in some patients, amygdala changes may occur before motor symptoms appear, and by the time of diagnosis, the volume may already have reached a plateau, resulting in non-significant differences between early- and late-stage groups. On the other hand, in the body-first subtype, amygdala involvement may occur later. Mixing patients with different subtypes but the same clinical stage can attenuate the correlation between amygdala volume and disease stage. A review (Horsager et al., 2022; Horsager and Borghammer, 2024) further supports our interpretation, noting that structural MRI volumetry may be less sensitive than molecular imaging or clinical features in distinguishing the two subtypes.

After controlling for age, sex, education, and ICV, right anterior amygdaloid area (AAA) volume showed a weak positive correlation with olfactory threshold (TT) and TDI total score in PD patients. However, these correlations were not statistically significant. Therefore, these findings do not support a specific role of the AAA in PD-related olfactory dysfunction. As such, these results cannot support a specific role of the AAA in PD-related olfactory dysfunction. Instead, they should be interpreted as preliminary exploratory findings that need to be confirmed in larger samples and independent cohorts.

The AAA is located in the anteromedial amygdala, belongs to the extension of the basolateral complex, receives monosynaptic projections from the olfactory bulb, is an important component of the primary olfactory cortex, and participates in olfactory emotional processing (Lane et al., 2020; Noto et al., 2021). Noto et al. suggested, through resting-state functional connectivity, that the anteromedial amygdala is involved in olfactory-related emotional learning and memory (Noto et al., 2021). Yang et al. confirmed high fiber connection density between the anterior cortical nucleus, medial nucleus, and periamygdaloid complex and the olfactory bulb (Yang et al., 2025). These anatomical and functional foundations provide a structural basis for the findings of this study.

The lack of significant correlation between the basolateral subregions (e.g., La, Ba, AB), which are closely related to AAA, and olfactory scores reflects functional differentiation within the amygdala: subregions that directly receive olfactory bulb input (e.g., AAA) have a more direct impact on olfactory function, while subregions primarily involved in emotional regulation (e.g., Ba, AB) have a relatively indirect relationship with olfactory sensory function, or may show correlation only at later stages.

The weak positive correlation between right AAA volume and olfactory scores observed in this study is generally consistent with previous neuroimaging findings, yet also shows some subregion-specific characteristics. In PD, Ay et al. (2023) found that bilateral CAT and superficial cortex-like region (sCLR) volumes were significantly reduced in PD patients with cognitive impairment, and left olfactory-related amygdala volume successfully discriminated cognitive status (Ay et al., 2023). However, the accuracy for olfactory-based grouping was only 64.8 and 61.1%, suggesting that changes in olfactory amygdala subregion volumes may be more closely associated with cognitive function than with pure olfactory scores, the finding consistent with our results. Studies on the AD spectrum further support the role of the amygdala in olfactory dysfunction: Cavedo et al. (2011) revealed using *in vivo* MRI that the amygdala is closely related to the olfactory circuit in AD, and Hagemeier et al. (2016) reported that left amygdala volume is significantly correlated with odor identification deficits in aMCI patients. Recent rs-fMRI studies provide complementary evidence from a functional connectivity perspective. Cieri et al. (2024) found persistently lower functional connectivity in olfactory-related brain regions (including the amygdala) in PD patients with cognitive impairment. Wang et al. (2022) further reported widespread reduced functional connectivity of the amygdala in PD patients with severe olfactory dysfunction, particularly involving connections with the inferior parietal lobule and lingual gyrus.

Partial rank correlation analysis revealed that the volume of the left medial subdivision (Me) of the amygdala was significantly positively correlated with MMSE scores in PD patients, suggesting that Me structural integrity may be associated with the maintenance of global cognitive function in PD. Notably, one-way ANOVA among the three groups (HC, ES, LS) showed no significant difference in left Me volume, which contrasts with the significant inter-group differences observed in the Ba, Co, AB, CAT, and AAA subregions. This dissociation—significant correlation but non-significant group difference—implies that left Me volume changes may not reflect stage-specific atrophy related to motor staging, but rather a continuous linear dimension associated with cognitive decline. In other words, Me volume may be more sensitive to inter-individual variability in cognitive performance than to coarse-grained motor staging. Its volumetric changes across the PD course are relatively subtle and may not reach detection threshold in group-wise staging comparisons; nevertheless, its linear relationship with MMSE robustly underscores the structural contribution of this nucleus to cognitive preservation. From an anatomical and functional perspective, Me is one of the most closely connected subregions to the olfactory system, receiving direct projections from the olfactory bulb and piriform cortex (Yang et al., 2025). Braak’s pathological staging theory indicates that the amygdala is affected relatively early in PD. Moreover, Me participates in emotional and cognitive integration via projections to limbic structures such as the bed nucleus of the stria terminalis (Lebow and Chen, 2016). Our finding is consistent with the hypothesis that olfactory dysfunction and cognitive impairment in PD may be linked through cholinergic dysfunction (Barrett et al., 2021; Pasquini et al., 2021; Bohnen et al., 2022), and the association between Me volume and MMSE may partially reflect the structural impact of cholinergic system degeneration on limbic networks.

Previous studies have largely focused on other amygdala subregions: Seo et al. (2025) found that the left basal nucleus (Ba) volume was significantly reduced in PD patients who developed cognitive impairment, and Zhang et al. reported diverse alterations across multiple subregions in PD-MCI patients. In contrast (Zhang et al., 2024), our study is the first to demonstrate a specific correlation between Me volume and MMSE in PD, independent of disease staging, further emphasizing the distinct structural roles of different amygdala subnuclei in PD-related cognitive impairment. Me structural integrity may serve as a potential continuous neuroimaging biomarker of cognitive status in PD. Future studies should incorporate multi-domain cognitive assessments and longitudinal follow-ups to further clarify the predictive value of Me volume changes for cognitive decline in PD.

## 5 Limitations

There are several potential limitations should be noted. First, limited sample size (only 25 late-stage PD patients), which may affect statistical power; some subregion volume differences were only at borderline significance after multiple comparison correction, requiring validation with larger samples. Second, the cross-sectional design does not allow direct observation of dynamic change trajectories within the same patient or determination of causality; longitudinal studies are needed in the future. Besides, Amygdala segmentation was based on 3T MRI; future studies could combine higher field strength MRI or histological validation to improve accuracy. Our volumetric analysis focused only on the amygdala, without including other key regions of the olfactory network. Recently updated parcellations of the primary olfactory cortex (Zhou et al., 2019) and the comprehensive Sense of Smell Atlas, which maps both cortical and subcortical components of the olfactory system (Gaviraghi et al., 2026), could provide valuable tools for future studies to integrate multi-regional analyses and better localize the specific role of the amygdala within the broader olfactory network, including patterns of lateralization. Finally, although this study employed a deep learning-based automated segmentation model combined with the FreeSurfer framework to minimize segmentation errors, partial volume effects and segmentation variability at 1 mm^3^ resolution may still affect the reliability of the measurements due to the small size of the amygdala subregions. Therefore, caution is needed when interpreting the results.

## 6 Conclusion

This study found subregion-selective atrophy of the amygdala in Parkinson’s disease patients, with the cortical nucleus and anterior amygdaloid area (AAA) affected early, and the accessory basal nucleus and cortico-amygdaloid transition area affected later, showing a right-lateralized pattern. The weak correlation between right AAA volume and olfactory function suggests a specific role of AAA in PD-related olfactory dysfunction. These findings provide novel insights into the structural basis of olfactory dysfunction in PD.

## Data Availability

The original contributions presented in the study are included in the article/supplementary material, further inquiries can be directed to the corresponding author.

## Footnotes

1
https://urp.united-imaging.com
